# *MTHFR* rs1801133 Polymorphism Is Associated With Liver Fibrosis Progression in Chronic Hepatitis C: A Retrospective Study

**DOI:** 10.3389/fmed.2020.582666

**Published:** 2020-11-13

**Authors:** Daniel Pineda-Tenor, Ana Zaida Gómez-Moreno, Juan José Sánchez-Ruano, Tomas Artaza-Varasa, Ana Virseda-Berdices, Amanda Fernández-Rodríguez, Pedro Molina Mendoza, María Ángeles Jiménez-Sousa, Salvador Resino

**Affiliations:** ^1^Servicio de Laboratorio Clínico, Hospital de Antequera, Málaga, Spain; ^2^Servicio de Digestivo, Hospital Virgen de la Salud, Toledo, Spain; ^3^Unidad de Infección Viral e Inmunidad, Centro Nacional de Microbiología, Instituto de Salud Carlos III, Majadahonda, Spain

**Keywords:** chronic hepatitis C, liver stiffness measure, hepatic fibrosis, cirrhosis, MTHFR (C677T), SNPs (single nucleotide polymorphism)

## Abstract

**Background:** The *MTHFR* (methylenetetrahydrofolate reductase) rs1801133 polymorphism leads to higher circulating levels of homocysteine, which is related to several liver diseases. We aimed to evaluate the relationship between *MTHFR* rs1801133 polymorphism and liver fibrosis progression in HCV-infected patients.

**Methods:** We conducted a preliminary retrospective cohort study in 208 non-cirrhotic HCV-infected patients. These subjects had at least two liver stiffness measurements (LSM), which were assessed using transient elastography, and no patient had cirrhosis at baseline. We analyzed the association between *MTHFR* rs1801133 and outcome variables using Generalized Linear Models.

**Results:** HCV-infected patients were 47 years old, around 54% were males, a low frequency of high alcohol intake (13.5%) or prior use of intravenous drugs (10.1%). A total of 26 patients developed cirrhosis (LSM1 ≥ 12.5) during a median follow-up of 46.6 months. The presence of the rs1801133 C allele showed an inverse association with the LSM2/LSM1 ratio (adjusted AMR = 0.90; 95%CI = 0.83–0.98; *p* = 0.020) and the cirrhosis progression (adjusted OR = 0.43; 95%CI = 0.19–0.95; *p* = 0.038). Besides, rs1801133 CT/CC genotype had an inverse association with the LSM2/LSM1 ratio (adjusted AMR = 0.80; 95%CI = 0.68–0.95; *p* = 0.009) and the cirrhosis progression (adjusted OR= 0.21; 95%CI = 0.06–0.74; *p* = 0.015).

**Conclusions:** *MTHFR* rs1801133 C allele carriers presented a diminished risk of liver fibrosis progression and development of cirrhosis than rs1801133 T allele carriers. This statement supports the hypothesis that *MTHFR* rs1801133 polymorphism appears to play a crucial role in chronic hepatitis C immunopathogenesis.

## Introduction

According to the world health organization, viral hepatitis is a significant public health problem that causes 1.34 million deaths per year due to chronic liver disease (720,000 by cirrhosis) and primary liver cancer (47,000 by hepatocellular carcinoma) (1). Globally, around 71 million people suffer chronic hepatitis C (CHC) and the development of the previously described events is frequent (2, 3), even after a sustained virological response (SVR) to treatment with direct-acting antivirals (DAAs) (4, 5). The pathogenic mechanisms involved in the progression of fibrosis and cirrhosis depends, among others, on the genetic background of individuals, including several single nucleotide polymorphisms (SNPs) (6, 7).

The staging of liver fibrosis provides essential clinical information that allows the adequate management and prognosis of CHC patients (8). The liver biopsy has been used to grade the necroinflammatory activity and to stage fibrosis, together with rating scales like METAVIR, which stratify fibrosis as: (i) F0, no fibrosis; (ii) F1, mild fibrosis; (iii) F2, significant fibrosis; (iv) F3, advanced fibrosis; and (v) F4, cirrhosis (9). Nevertheless, non-invasive approaches, as the transient elastography or FibroScan, have been widely used to the liver fibrosis assessment, with excellent accuracy in advanced fibrosis and cirrhosis (10). In this context, the evaluation of liver stiffness measurement (LSM), an intrinsic physical property of liver parenchyma, provides quantitative data that correlates with fibrosis stage in CHC (11).

The methylenetetrahydrofolate reductase (*MTHFR*) gene encodes an enzyme that plays an important role in the folate metabolism, allowing the conversion from homocysteine to methionine (12). The rs1801133 SNP (also named C677T) is a non-synonymous variant A (Ala) > V (Val) (missense variant) (13). The substitution A > V in the aminoacid 222 (Ala222Val) produces a decrease of the activity of MTHFR protein and an elevation of plasma homocysteine levels (13). Hyper-homocysteinemia was related to several diseases, including hepatocellular carcinoma, steatosis, and cirrhosis (14–16), and also the development of liver fibrosis in CHC (17). There are a few articles published about *MTHFR* rs1801133 in CHC patients. *MTHFR* C677T polymorphism has been related to hepatic steatosis (18) and development of liver fibrosis (16, 17), but no association was found in other articles in patients infected with HCV (19–21). Additionally, this polymorphism could also be related to a direct profibrogenic effect, modifying the action of proteins implicated in the degradation of collagen (22).

The main objective in the current study was to investigate the association of *MTHFR* rs1801133 polymorphism with the progression of liver fibrosis and cirrhosis development, evaluated by LSM, in patients with CHC.

## Materials and Methods

### Study Population

A preliminary retrospective study was performed in 208 patients from the Hospital Virgen de la Salud (Toledo, Spain). All patients suffered from chronic hepatitis C and were enrolled between 2008 and 2016, as previously described (see Supplementary Figure 1) (23).

We selected the patients according to these criteria: (i) available DNA sample; (ii) detectable plasma HCV RNA at baseline and during follow-up; and (iii) baseline LSM (LSM1) and final LSM (LSM2) available with a separation of 12 months at least. Regarding the exclusion criteria, we considered: (i) liver cirrhosis at baseline (F4; LSM1 ≥ 12.5 kPa); (ii) coinfection with human immunodeficiency virus or hepatitis B virus; and (iii) autoimmune liver disease.

The study was conducted with the consent of all patients and following the 1975 Declaration of Helsinki. It was approved by the Institutional Review Board of the Instituto de Salud Carlos III (“Comité de Ética de la Investigación y Bienestar Animal” –April 4, 2013).

### Clinical Data

As described previously (24), clinical and epidemiological data were collected from medical records. These data included virological, demographic, clinical, and laboratory data. Clinical guidelines available at that time (25, 26) were followed to perform the clinical management of patients during the follow-up.

At baseline, we only included non-responder patients (patients treated for HCV infection before the study), although it was possible to administrate the HCV therapy before or after being included in the study. During the follow-up, the monitoring was stopped when a patient started the HCV therapy and obtained an SVR.

### DNA Genotyping

Genomic DNA obtained from 200 microliters of peripheral blood was extracted using the QIAsymphony DNA Mini Kit (Qiagen, Hilden, Germany). *MTHFR rs1801133* polymorphism was genotyped at the CeGen (Spanish National Genotyping Center; http://www.cegen.org/). Agena Bioscience's MassARRAY platform (San Diego, CA, USA) and the iPLEX® Gold assay design system were used according to the method described by Gabriel et al. (27).

### Evaluation of Liver Fibrosis

The hepatic fibrosis was evaluated using the transient elastography (FibroScan, Echosens, Paris, France), by a trained hepatologist and with a single machine, as we previously described (24). LSM has a range of 2.5 to 75 kilopascals (kPa). When the interquartile-range-to-median ratio for at least 10 successful measurements was <0.30, it was considered reliable. The cut-offs of LSM proposed by Castera et al. were followed for the stratification of patients: (i) <7.1 kPa (F0–F1—absence or mild fibrosis); (ii) 7.1–9.4 kPa (F2—significant fibrosis); (iii) 9.5-12.4 kPa (F3—advanced fibrosis); and (iv) ≥12.5 kPa (F4—cirrhosis) (28).

### Outcome Variable

We analyzed how LSM values changed during the follow-up, considering: (i) the date of the first LSM (LSM1) and (ii) the date of the last LSM (LSM2), or the date when the HCV therapy started in responder patients who cleared HCV infection. For this propose, we consider two outcome variables: (1) LSM2/LSM1 ratio; (2) the cirrhosis progression (F4; LSM ≥ 12.5 kPa) measured as +1 [if a patient with LSM <12.5 kPa (F ≤ 3) changed to LSM ≥12.5 kPa (F4)] or 0 (if a patient with F ≤ 3 did not evolve to F4).

### Statistical Analysis

To compare independent groups, we used the Mann-Whitney *U* test for continuous variables and the Chi-square test or Fisher's exact test for categorical variables. In the case of paired measurements, we used the Sign test for categorical variables and the Wilcoxon signed-rank test for continuous variables.

We used Generalized Linear Models (GLM) according to recessive, dominant, and additive inheritance models for the genetic association study with the aim of comparing the outcome variables according to *MTHFR* rs1801133. First, we used a GLM with a gamma distribution (log-link) to analyze continuous variables (LSM2/LSM1 ratio) and a GLM with a binomial distribution (logit-link) to analyze dichotomous variables (progression to cirrhosis). These tests provide the arithmetic mean ratio (AMR) or difference between groups, and the odds ratio (OR) or probability of occurrence of an event. The most relevant patient characteristics were used to adjust the GLM tests: gender, age, diabetes, high alcohol intake, injection drug use, time since HCV diagnosis, HCV genotype, baseline LSM, time of follow-up, HCV antiviral therapy before baseline and during the follow-up (patients who failed therapy), and other SNPs previously described in this study population (*PNPLA3* rs738409 (29), *MERTK* rs4374383 (30), *IL7RA* rs6897932 (24), and *DARC* rs12075 (23). To avoid overfitting the statistical models, we made a previous selection of covariables with the Stepwise algorithm, retaining covariables with a *p*-value <0.20 at each step.

For all statistical tests, we used the Stata 15.0 (StataCorp, Texas, USA) and SPSS 24.0 (SPSS INC, Chicago, IL, USA). Statistical significance was defined as *p* < 0.05 and all *p*-values were two-tailed.

## Results

### Characteristics of the Patients

The baseline characteristics of our study population are described in Table 1. HCV-infected patients were 47 years old, around 54% were males, a low frequency of high alcohol intake (13.5%) or prior use of intravenous drugs (10.1%). HCV genotype 1 was the predominant (85.3% of patients), 22.6% of patients previously failed the interferon therapy, and 71.6% of patients had LSM <7.1 kPa. Concerning rs1801133 genotypes, 29 patients were TT genotype, 112 were CT genotype, and 67 were CC genotype. No significant differences in baseline characteristics were found among rs1801133 genotypes, except for prior injection drug use (*p* = 0.008).

**Table 1:** Epidemiological and clinical characteristics of HCV-infected patients at baseline.

		***MTHFR*** **rs1801133 polymorphism**			
**Characteristic**	**All patients**	**TT**	**CT**	**CC**	***P*-value**
No.	208	29	112	67	
Male	112 (53.8%)	14 (48.33%)	65 (58%)	33(49.3%)	0.423
Age (years)	47.1(41.5; 57.6)	46.96 (39.5; 57.3)	46.85 (41.3; 55.4)	48.2 (42.6; 60.8)	0.988
Time of HCV infection (years)	8.2 (3.2; 13.2)	7.8 (3.3; 11.9)	8.1 (2.9; 13.3)	9 (2.7; 13.3)	0.959
High alcohol intake	28 (13.5%)	1 (3.4%)	20 (17.9%)	7 (10.4%)	0.087
Prior injection drug use	21 (10.1%)	1 (3.4%)	18 (16.1%)	2 (3%)	**0.008**
HCV genotype (*n* = 204)					
1	174 (85.3%)	26 (92.9%)	90 (82.6%)	58 (86.6%)	0.366
3	14 (6.9%)	1 (3.6%)	9 (8.3%)	4 (6%)	0.641
4	15 (7.4%)	1 (3.6%)	9 (8.3%)	5 (7.5%)	0.698
5	1 (0.5%)	0 (0.0%)	1 (0.9%)	0 (0%)	-
Prior failed IFN therapy	47 (22.6%)	6 (20.7%)	26 (23.2%)	15 (22.4%)	0.958
Baseline LSM (kPa)	6.1 (5.2; 7.7)	6.6 (5.2; 7.5)	6.1 (5.2; 7.7)	5.9 (5.1; 7.7)	0.961
F0–F1 (<7.1 kPa)	149 (71.6%)	21 (72.4%)	79 (70.5%)	49 (73.1%)	0.928
F2 (7.1–9.4 kPa)	38 (18.3%)	5 (18.2%)	22 (19.6%)	11 (16.4%)	0.873
F3 (9.5–12.4 kPa)	21 (10.1%)	3 (6.8%)	11 (9.8%)	7 (10.4%)	0.904

Statistics: Values are expressed as absolute numbers (%) or median (percentile 25; percentile 75). P-values were estimated with Chi-square test for categorical variables and non-parametric Mann-Whitney U test for continuous variables.

HCV, hepatitis C virus; IFN, interferon; kPa, kilopascal; LSM, liver stiffness measure; MTHFR, methylenetetrahydrofolate reductase.

*Significant differences are shown in bold*.

### Characteristics of *MTHFR* rs1801133 Polymorphism

Table 2 describes the allelic and genotypic frequencies of the rs1801133 SNP, which showed <5% of missing values, was in Hardy-Weinberg equilibrium (*p* = 0.252) and had a minimum allele frequency more than 40%. We compared the genetic frequencies between patients included in this study and the Iberian population in Spain (IBS), a population of healthy subjects published by the 1,000 Genomes Project website (http://www.1000genomes.org/home). No significant differences were found for alleles (*p* = 0.940) or genotypes (*p* = 0.665).

**Table 2:** Allelic and genotypic frequencies and Hardy Weinberg Equilibrium test for *MTHFR rs1801133* polymorphism in HCV-infected patients compared to Iberian population (data from 1,000 Genomes Project Phase 3) (http://grch37.ensembl.org/index.html).

		**HCV cohort**	**IBS group**	***P*-value**
No.		208	107	
Alleles	T	141 (44.1%)	95 (44.4%)	0.940
	C	179 (55.9%)	119 (55.6%)	
Genotype	TT	29 (13.9%)	18 (16.8)	0.665
	CT	112 (53.8%)	59 (55.1%)	
	CC	67 (32.2%)	30 (28.0%)	
HWE (*p*-value)		0.252	0.241	

Statistics: Values are expressed as absolute numbers (%) p-values were estimated with Chi-squared test.

*HCV, hepatitis C virus; HWE, Hardy Weinberg Equilibrium; IBS, Iberian populations in Spain; MTHFR, methylenetetrahydrofolate reductase*.

### *MTHFR* rs1801133 SNP and Related Liver Fibrosis Progression

The mean follow-up time (the period between the LSM1 and the LSM2) for all patients was of 46.6 months. In this context, we found a decrease in the proportion of patients with a low stage of fibrosis (F0–F1; *p* < 0.001), whereas both LSM values and the rate of patients who developed an F4 stage raised (*p* < 0.001) (Table 3).

**Table 3:** Clinical characteristics related to hepatic fibrosis in patients with chronic hepatitis C during the follow-up.

	**All Patients**		
**Characteristic**	**Baseline**	**End**	***P*-value**
LSM (kPa)	6.1 (5.2; 7.7)	6.8 (5.5; 9.4)	**<0.001**
F0–F1 (<7.1 kPa)	149 (71.6%)	110 (52.9%)	**<0.001**
F2 (7.1–9.4 kPa)	38 (18.3%)	47 (22.6%)	0.382
F3 (9.5–12.4 kPa)	21 (10.1%)	25 (12%)	0.571
F4 (≥12.5 kPa)	0 (0%)	26 (12.5%)	**<0.001**

Statistics: Values are expressed as absolute numbers (%) or median (percentile 25; percentile 75). P-values were calculated with Sign test for categorical variables and nonparametric Wilcoxon test for continuous variables.

HCV, hepatitis C virus; kPa, kilopascal; LSM, liver stiffness measurement; F ≥ 2, significant fibrosis; F ≥ 3, advanced fibrosis; F4, cirrhosis.

*Significant differences are shown in bold*.

We did not find significant differences among rs1801133 genotypes in the time intervals between the LSM1 and the LSM2 (48.5 months in TT genotype, 46.9 months in CT genotype, and 45.5 months in CC genotype; *p* = 0.921). We observed decreased values of the LSM2/LSM1 ratio and the rate of cirrhosis progression in rs1801133 T allele carriers (Table 4). Moreover, we analyzed the association between *MTHFR* rs1801133 polymorphism and liver fibrosis/cirrhosis progression through multivariate GLMs (Table 4, full description in Supplementary Table 1). The presence of the rs1801133 C allele showed an inverse association with the LSM2/LSM1 ratio (adjusted AMR = 0.90; 95%CI = 0.83–0.98; *p* = 0.020) and the cirrhosis progression (adjusted OR = 0.43; 95%CI = 0.19–0.95; *p* = 0.038). Besides, rs1801133 CT/CC genotype had an inverse association with the LSM2/LSM1 ratio (adjusted AMR = 0.80; 95%CI = 0.68–0.95; *p* = 0.009) and the cirrhosis progression (adjusted OR = 0.21; 95%CI = 0.06–0.74; *p* = 0.015) (Table 4).

**Table 4:** Association between *MTHFR* rs1801133 polymorphism and progression of liver fibrosis in patients with chronic hepatitis C (longitudinal analysis).

	***MTHFR*** **rs1801133 genotypes**			**Unadjusted**		**Adjusted**	
**Outcome**	**TT**	**CT**	**CC**	**AMR (95%CI)**	***P*^**a**^**	**aAMR (95%CI)**	***P*^**b**^**
**LSM2/LSM1**							
Additive	1.19 (1.00; 1.43)	1.14 (0.95; 1.44)	1.10 (0.89; 1.34)	0.85 (0.77; 0.94)	**0.001**	0.90 (0.83; 0.98)	**0.020**
Dominant	1.19 (1.00; 1.43)	1.13 (0.95; 1.43)		0.77 (0.64; 0.93)	**0.006**	0.80 (0.68; 0.95)	**0.009**
**Progression to F4**	**TT**	**CT**	**CC**	**OR (95%CI)**	***P***^**a**^	**aOR (95%CI)**	***P***^**b**^
Additive	7 (24.10%)	13 (11.60%)	6 (9.00%)	0.55 (0.29; 1.04)	0.069	0.43 (0.19; 0.95)	**0.038**
Dominant	7 (24.10%)	19 (10.60%)		0.37 (0.14; 0.99)	**0.047**	0.21 (0.06; 0.74)	**0.015**

Statistics: Values expressed as absolute numbers (%), median (percentile 25; percentile 75), arithmetic mean ratio (AMR), odds ratio (OR), and 95% of confidence interval (95%CI).

a P-values were calculated by univariate regression;

b P-values were calculated by multivariate regression adjusted by the most important clinical and epidemiological characteristics (see statistical analysis section). Significant differences are shown in bold.

*aAMR, adjusted arithmetic mean ratio; aOR, adjusted odds ratio; 95%CI, 95% confidence interval; p-value, level of significance; LSM, liver stiffness measure; F4, cirrhosis; MTHFR, methylenetetrahydrofolate reductase*.

## Discussion

The present study focused on the potential relationship of *MTHFR* rs1801133 polymorphism and the development of liver fibrosis and cirrhosis on HCV-infected patients, using two LSM values with an interval of at least 12 months. We observed that CT/CC genotype was related to a decreased risk of progression of liver fibrosis and the occurrence of cirrhosis.

The C677T genetic substitution at the *MTHFR* gene results in the Ala222Val replacement in the MTHFR protein, resulting in a thermolabile variant associated with lower activity, and therefore, higher circulating levels of homocysteine (13). Moreover, *MTHFR* rs1801133 SNP is in high linkage disequilibrium (LD) with other *MTHFR* SNPs, such as A1298C (rs1801131), also related to a decrease in MTHFR activity (31). The LD between the two SNPs is strong in the Spanish population (coefficient of LD = 0.98) (32), so we think that our results may be extrapolated to the A1298C variant and other *MTHFR* SNPs in LD with rs1801133.

The *MTHFR* rs1801133 SNP was previously linked to a long list of conditions and diseases, such as bone disorders, cardiovascular disease, thrombosis, neurological/neuropsychiatric conditions (33), pre-eclampsia, diabetes mellitus (34), longevity (35), and several types of neoplasia (36). Regarding liver diseases, the *MTHFR* rs1801133 polymorphism is related to altered lipid metabolism (37), which would contribute to the development of steatosis and fibrosis in HCV-infected patients (38), as well as the development of cirrhosis (14, 39–41). Furthermore, in CHC, the *MTHFR* rs1801133 variant has also been linked to liver fibrosis/cirrhosis (16, 17). Toniutto et al. described that recipients with the presence of *MTHFR* rs1801133 TT homozygote evolved with more frequency to a significant fibrosis degree during recurrent hepatitis C after liver transplantation (16). Similar to this, Adinolfi et al. described that the T allele is related to a higher prevalence of steatosis, accelerating the fibrosis development and liver disease progression (17). These analyses support that the *MTHFR* rs1801133 polymorphism and the subsequent hyperhomocysteinemia are associated with liver fibrosis among HCV-infected patients. However, there are also other articles that found no association between the *MTHFR* rs1801133 variant and liver fibrosis/cirrhosis (19–21). These articles, both those that showed an association and those that did not, had a cross-sectional design, and fibrosis was evaluated by biopsy. Our article, by contrast, had a longitudinal design that provides robustness to our data, and liver fibrosis was evaluated by transient elastography, which has excellent accuracy for cirrhosis diagnosis.

Other issues should be considered for the correct interpretation of the data. Firstly, we performed a retrospective study, which could induce ascertainment and selection biases. Secondly, the low sample size per group could limit the statistical power of the tests performed and increasing the rate of false positives. Therefore, further studies should be conducted to corroborate our preliminary findings on the potential use of *MTHFR* rs1801133 SNP as a predictive marker of liver fibrosis/cirrhosis progression in HCV-infected patients. Thirdly, the follow-up time (between LSM1 and LSM2) varied between different subjects, but 75% of the patients had more than 28 months of follow-up, and globally, all patients presented more than 12 months of follow-up. Besides, the time of follow-up in patients stratified by *MTHFR* rs1801133 genotypes was comparable. Fourthly, due to the retrospective design of our investigation, we did not take into account some important clinical variables, including obesity, abdominal ultrasound, metabolic syndrome, and pathological study of the liver (fibrosis, necroinflammation, and steatosis), among others; and biomarkers, such as HCV viral load, transaminases, platelet counts, APRI score, and FIB4 index, among others. We did not have access to these data at the time of the LSM. Moreover, we had not plasma samples available to measure concentrations of homocysteine. Finally, we included in the study more than 20% non-responders to previous interferon therapy, but this HCV therapy does not seem to protect against the progression of CHC in the long term studies (42).

## Conclusion

In summary, in this preliminary study, our data suggest an association between *MTHFR* rs1801133 SNP and the progression of liver fibrosis and the development of cirrhosis in HCV-infected patients. Specifically, *MTHFR* rs1801133 C allele carriers presented a diminished risk of liver fibrosis progression and development of cirrhosis than rs1801133 T allele carriers. Further studies with higher numbers of patients would be needed to confirm the role of *MTHFR* in the immune-pathogenesis of CHC.

## Data Availability Statement

The datasets used and analyzed during the current study may be made available by the corresponding author upon reasonable request.

## Ethics Statement

The studies involving human participants were reviewed and approved by the study was conducted following the 1975 Declaration of Helsinki. The Institutional Review Board of the Instituto de Salud Carlos III (Comité de Ética de la Investigación y Bienestar Animal 04/04/2013) approved the study, and all patients gave their consent for the study. The patients/participants provided their written informed consent to participate in this study.

## Author Contributions

MJ-S and SR: funding body, study concept and design. AG-M, MJ-S, JS-R, and TA-V: patients' selection and clinical data acquisition. DP-T, MJ-S, and AG-M: sample preparation, DNA isolation and genotyping. DP-T, MJ-S, AV-B, and SR: statistical analysis and interpretation of data. DP-T, MJ-S, and SR: writing of the manuscript. DP-T, AF-R, and PM: critical revision of the manuscript for relevant intellectual content. SR: supervision and visualization. All authors contributed to the article and approved the submitted version.

## Conflict of Interest

The authors declare that the research was conducted in the absence of any commercial or financial relationships that could be construed as a potential conflict of interest.

## Abbreviations

CHC: chronic hepatitis C
HCV: hepatitis C virus
DAAs: direct-acting antivirals
SNPs: single nucleotide polymorphisms
LSM: liver stiffness measurement
MTHFR: methylenetetrahydrofolate reductase; F4
LSM1 ≥ 12.5 kPa: cirrhosis
LSM1: baseline LSM
LSM2: final LSM
ΔLSM: increase of LSM
GLM: generalized linear models
AMR: arithmetic mean ratio
OR: odds ratio
SPSS: statistical package for the social sciences
IBS: Iberian population in Spain.
