# Supplementary figures and images for: MTHFR rs1801133 Polymorphism Is Associated With Liver Fibrosis Progression in Chronic Hepatitis C: A Retrospective Study
Source: Front Med (Lausanne). 2020 Nov 13;7:582666. doi: 10.3389/fmed.2020.582666 (PMC7691664; doi:10.3389/fmed.2020.582666)

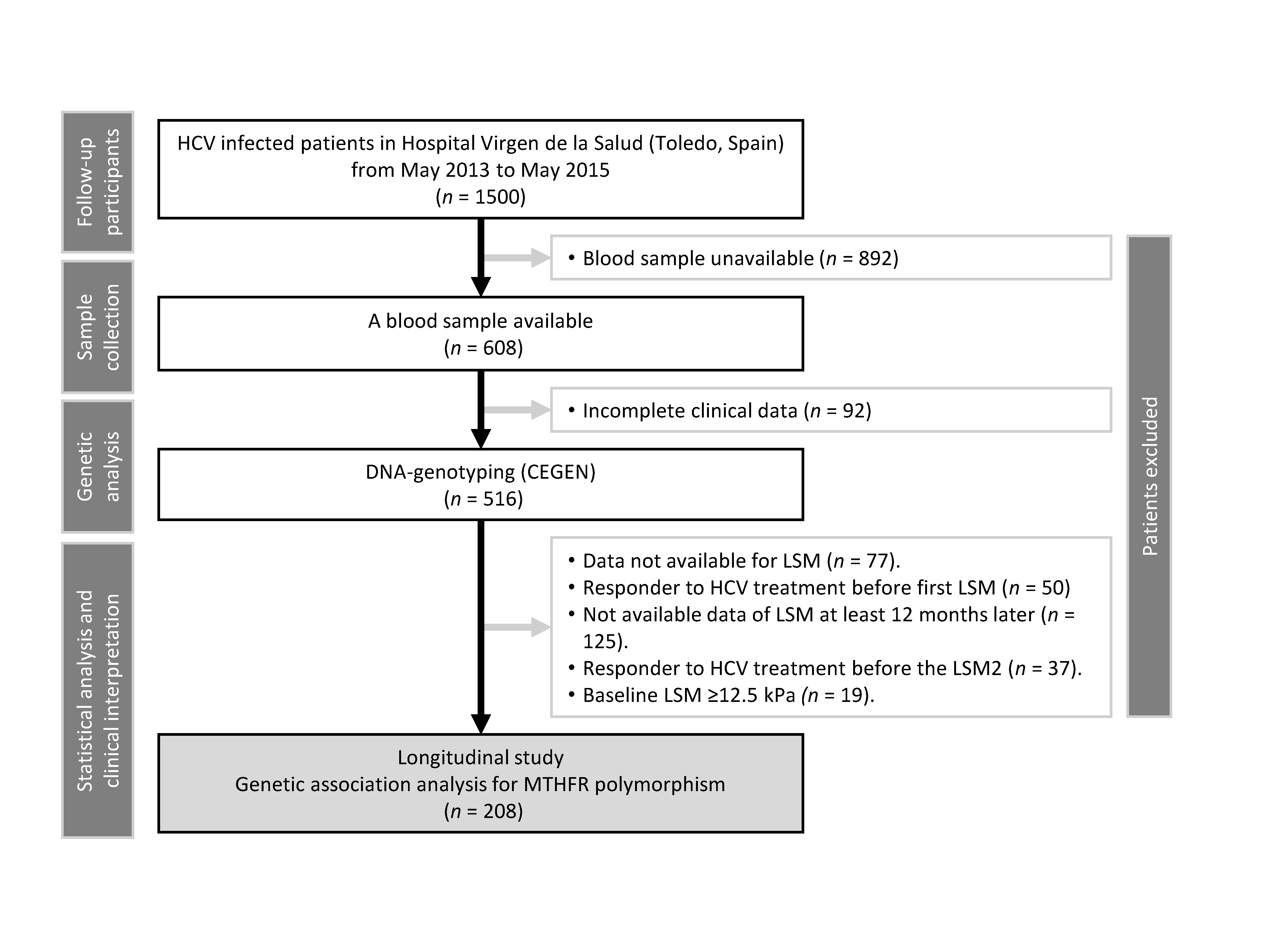

Supplement: Supplementary file 1 [file Image_1.TIF]
