# Supplementary material for: MTHFR rs1801133 Polymorphism Is Associated With Liver Fibrosis Progression in Chronic Hepatitis C: A Retrospective Study
Source: Front Med (Lausanne). 2020 Nov 13;7:582666. doi: 10.3389/fmed.2020.582666 (PMC7691664; doi:10.3389/fmed.2020.582666)
Supplement: Supplementary file 2 [file Data_Sheet_1.PDF]

Supplementary Table 1. Adjusted association between *MTHFR* rs1801133 polymorphism and liver fibrosis/cirrhosis progression in HCV-infected patients.

		95% CI		
**A) Ratio LSM2/LSM1**	**aAMR**	**Low**	**up**	**p-value**
**Additive**				
MTHFR rs1801133 (G)	0.90	0.83	0.98	**0.020**
Gender (male)	1.16	1.03	1.30	**0.015**
Age (years)	1.00	1.00	1.01	0.136
High alcohol intake	1.17	0.98	1.38	0.079
Baseline LSM (kPa)	0.94	0.91	0.96	**<0.001**
HCV therapy during follow-up *	1.21	1.07	1.37	**0.002**
MERTK rs4374383 (G)	1.12	1.02	1.22	**0.012**
IL7RA rs6897932 (T)	1.16	1.05	1.28	**0.005**
DARC rs12075 (G)	0.92	0.85	1.00	**0.047**
PNPLA3 rs738409 (G)	1.11	1.02	1.21	**0.020**
**Dominant**				
MTHFR rs1801133 (AG/GG)	0.80	0.68	0.95	**0.009**
Gender (male)	1.16	1.03	1.30	**0.011**
Age (years)	1.00	1.00	1.01	0.137
High alcohol intake	1.21	1.02	1.44	**0.028**
Baseline LSM (kPa)	0.94	0.91	0.97	**<0.001**
Time of follow-up (months)	1.00	1.00	1.01	0.177
HCV therapy during follow-up *	1.23	1.09	1.38	**0.001**
MERTK rs4374383 (G)	1.13	1.03	1.23	**0.007**
IL7RA rs6897932 (T)	1.15	1.04	1.28	**0.005**
DARC rs12075 (G)	0.93	0.85	1.00	0.064
PNPLA3 rs738409 (G)	1.12	1.03	1.23	**0.010**

		95% CI		
**B) Cirrhosis progression (F4)**	**aOR**	**Low**	**up**	**p-value**
**Additive**				
MTHFR rs1801133 (G)	0.43	0.19	0.95	**0.038**
Gender (male)	3.10	1.01	9.53	**0.048**
Age (years)	1.06	1.00	1.12	**0.040**
Baseline LSM (kPa)	1.72	1.30	2.27	**<0.001**
HCV therapy during follow-up *	3.46	1.20	10.01	**0.022**
MERTK rs4374383 (G)	2.28	0.96	5.45	0.063
DARC rs12075 (G)	0.46	0.20	1.06	0.067
PNPLA3 rs738409 (G)	2.55	1.13	5.72	**0.024**
**Dominant**				
MTHFR rs1801133 (AG/GG)	0.21	0.06	0.74	**0.015**
Gender (male)	3.04	0.99	9.38	0.053
Age (years)	1.06	1.00	1.13	**0.039**
Baseline LSM (kPa)	1.72	1.30	2.27	**<0.001**
HCV therapy during follow-up *	3.40	1.16	9.97	**0.025**
MERTK rs4374383 (G)	2.34	0.93	5.91	0.071

IL7RA rs6897932 (T)	1.81	0.73	4.46	0.199
DARC rs12075 (G)	0.51	0.22	1.20	0.122
PNPLA3 rs738409 (G)	2.69	1.16	6.25	**0.021**

(*), Only patients who failed therapy were included.

Statistics: data were calculated by multivariate regression adjusted by the most important clinical and epidemiological characteristics (see **statistical analysis** section). Significant differences are shown in bold.

Abbreviations: aAMR, adjusted arithmetic mean ratio; aOR, adjusted odds ratio; 95%CI, 95% confidence interval; p-value, level of significance; LSM, liver stiffness measure; kPa, F4, cirrhosis; MTHFR, methylenetetrahydrofolate reductase.
